# A Giant Thoracic *ALK*‐Rearranged Mesenchymal Neoplasm in a Child

**DOI:** 10.1002/cnr2.70489

**Published:** 2026-02-11

**Authors:** Sheng Gao, Junhua Wu, Jun Fan, Xiaona Chang, Bo Huang, Danju Luo, Jun He, Heshui Shi, Xiaochuan Dong, Xiu Nie

**Affiliations:** ^1^ Department of Pathology Union Hospital, Tongji Medical College, Huazhong University of Science and Technology Wuhan Hubei China; ^2^ Department of Radiology Union Hospital, Tongji Medical College, Huazhong University of Science and Technology Wuhan Hubei China

**Keywords:** alectinib, *ALK*‐rearranged mesenchymal neoplasm, *PLEKHH2*::*ALK* fusion, thoracic

## Abstract

**Background:**

Mesenchymal neoplasms characterized by *ALK* fusions mainly include inflammatory myofibroblastic tumors (IMTs) and epithelioid fibrous histiocytomas (EFHs). More recently, *ALK*‐rearranged mesenchymal tumors that are not IMTs or EFHs, characterized by S100 and CD34 coexpression, have been reported in a few small series and isolated case reports. The neoplasms present a broad clinicopathological spectrum and variable biological behavior.

**Case Presentation:**

Here, we report the case of an 11‐year‐old girl with a giant mesenchymal neoplasm in her left thoracic cavity. Pathological biopsy revealed that the tumor was composed of monomorphic spindle cells arranged in a fascicular growth pattern with extensive necrosis and coexpression of S100 and CD34; subsequently, *PLEKHH2*::*ALK* fusion was identified via next‐generation sequencing (NGS). The patient underwent tumor resection via thoracoscopy. The specimen from radical resection indicated that the tumor was heterogeneous. Some tumor cells showed moderate to severe atypia with increased mitosis and necrosis, suggesting that the neoplasm had overtly malignant features and may be associated with an aggressive clinical course. The patient developed brain metastasis 3 months after surgery and subsequently responded well to targeted therapy with the ALK inhibitor alectinib.

**Conclusions:**

Our findings indicate that *ALK*‐rearranged mesenchymal neoplasms with fibrosarcoma‐like features, particularly those associated with elevated mitotic activity or tumor necrosis, should be classified as high grade in pathology reports. In addition, this case also demonstrated that neoadjuvant therapy may be a better treatment strategy compared to upfront surgery for *ALK*‐rearranged mesenchymal neoplasms with a relatively high tumor burden.

**Abbreviations**
*ALK*
: anaplastic lymphoma kinase
CT: computed tomography
EFHs: epithelioid fibrous histiocytomas
EIMSs: epithelioid inflammatory myofibroblastic sarcomas
FISH: fluorescence in situ hybridization
FNCLCC: Federation of Cancer Centers Sarcoma Group
HPFs: high‐power fields
IMTs: inflammatory myofibroblastic tumors
MFs: mitotic figures
MPNST: malignant peripheral nerve sheath tumor
MRI: magnetic resonance imaging
NGS: next‐generation sequencing
PD: progressive disease
PR: partial response

## 1 Introduction

The anaplastic lymphoma kinase (*ALK*) gene, located at chromosomal band 2p23, encodes a receptor tyrosine kinase (RTK) proto‐oncogene. The ALK fusion protein is predominantly activated through genetic rearrangements, leading to its constitutive expression and subsequent induction of oncogenic transformation [1, 2, 3, 4, 5, 6]. The subsets of mesenchymal tumors harboring *ALK* fusions mainly include inflammatory myofibroblastic tumors (IMTs), epithelioid inflammatory myofibroblastic sarcomas (EIMSs), and epithelioid fibrous histiocytomas (EFHs). In 2016, the first case of an *ALK*‐rearranged mesenchymal neoplasm (not identified as an IMT/EIMS or EFH) was reported by Agaram et al. [7]. Recently, a series of *ALK*‐rearranged mesenchymal neoplasms characterized by *ALK* fusions, CD34/S100 coexpression with negative SOX10, and a broad spectrum of histomorphology and histological grading have been reported [8]. The tumors were found in diverse locations, including superficial and deep soft tissue from the extremities, trunk, nasal cavity, and abdominal and visceral organs (kidney, urethra, and brain) [9, 10, 11, 12, 13, 14, 15, 16, 17, 18, 19, 20, 21, 22, 23, 24]. Histologically, *ALK*‐rearranged mesenchymal neoplasms present a wide range of histological features with diverse architectures and variable cellularity and cellular morphology sets, from spindled cells or oval cells to epithelioid cells, which are embedded in myxoid, myxohyaline, or collagenous stroma. The clinical biological behavior of *ALK*‐rearranged mesenchymal neoplasms has not been fully established. The majority of previously reported patients with available follow‐up data showed no tumor recurrence or metastasis following surgical resection, but a small subset exhibited aggressive disease progression, including local recurrence and distant metastasis [2, 3, 8, 22, 23, 24, 25]. As patients with these neoplasms may benefit from ALK inhibitor‐targeted therapy, which is now widely available for clinical use, accurate diagnosis of this entity is of clinical relevance.

Here, we present the case of an 11‐year‐old girl with a giant mesenchymal neoplasm in her left thoracic cavity, confirmed by biopsy as being an *ALK*‐rearranged mesenchymal tumor, who developed brain metastasis after surgery and subsequently responded well to targeted therapy with the ALK inhibitor alectinib. To our knowledge, this represents the first reported case in a child of a primary thoracic *ALK*‐rearranged mesenchymal neoplasm (outside of IMT/EIMS and EFH).

## 2 Case Presentation

**FIGURE 1:**
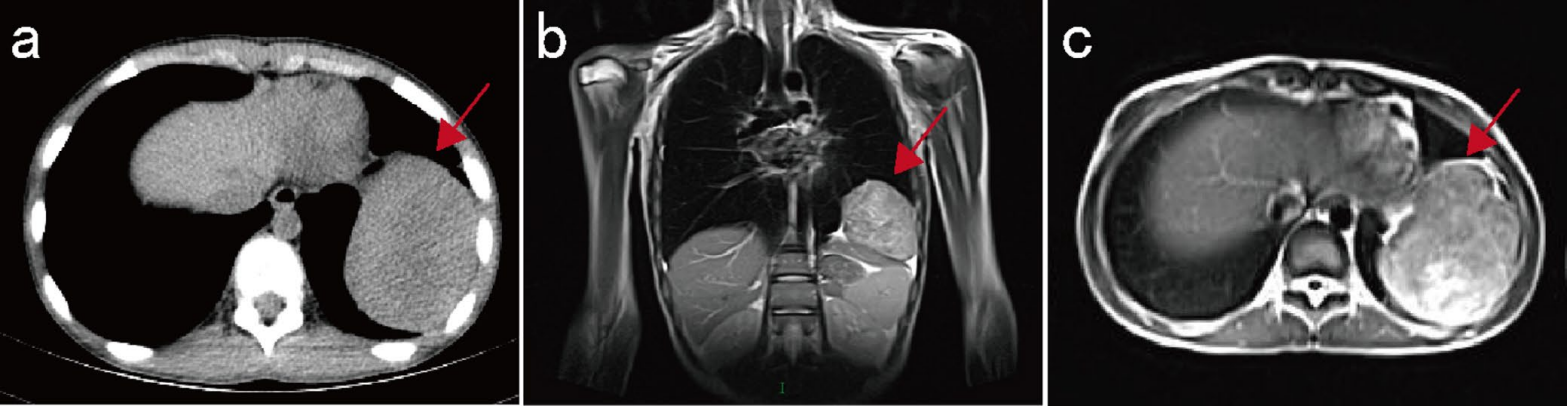
CT and MRI images of the thoracic cavity. (a) Transverse position CT image showing an uneven density and clear border mass located in the left thoracic cavity that is approximately 68 × 88 × 75 mm in size. (b, c) MRI revealed a well‐defined, heterogeneous soft tissue mass shadow located in the left thoracic cavity with localized areas of necrosis and mild compression of the diaphragm. (b) Coronal position, (c) transverse position. Red arrows indicate the mass shadow.

In May 2024, an 11‐year‐old girl with intermittent fever and joint swelling for over a week was admitted to Union Hospital, Tongji Medical College, Huazhong University of Science and Technology. A subsequent chest computed tomography (CT) scan revealed a giant uneven density mass located in the left thoracic cavity that was approximately 68 × 88 × 75 mm in size (Figure 1a). Contrast‐enhanced chest magnetic resonance imaging (MRI) revealed a well‐defined soft tissue mass shadow in the left thoracic cavity, with heterogeneous signal intensity, localized areas of necrosis, and mild compression of the diaphragm (Figure 1b,c).

**FIGURE 2:**
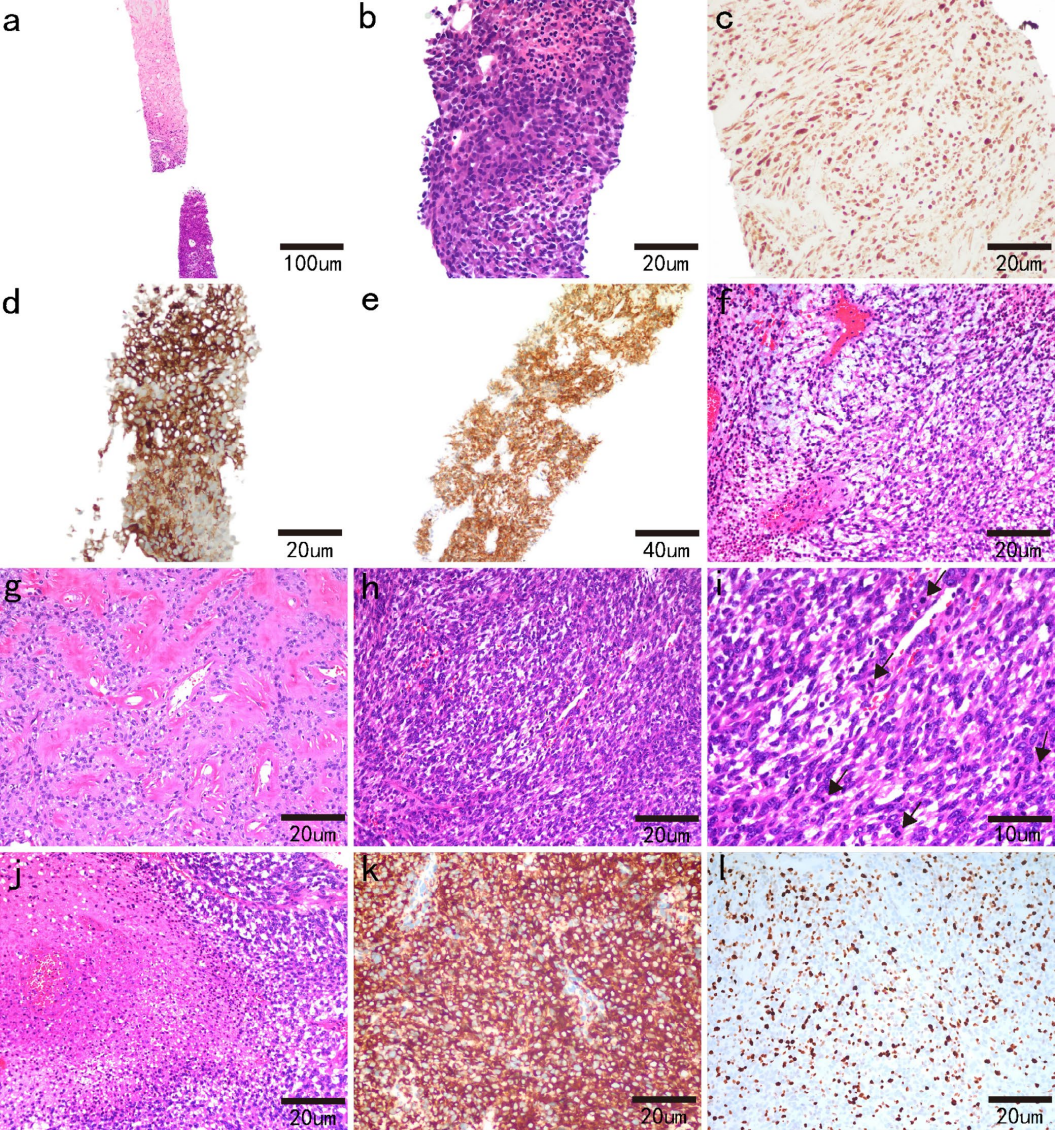
Microscopy image of the tumor. In the needle biopsy specimen (a–e), microscopic examination revealed a neoplastic lesion with extensive necrosis (a, 40×). The tumor consisted of spindle‐shaped mesenchymal cells and inflammatory infiltration under a high‐power field (b, 200×). The tumor cells were diffusely positive for S100 (c, 200×), CD34 (d, 200×), and ALK‐1A4 (e, 100×). Microscopy images of surgical tumor samples (f–l). Myxomatous stroma (f, 200×), focal hyalinized stroma, and perivascular hyalinization (g, 200×) were observed. In some areas, there was higher cellularity and moderate to severe cell pleomorphism (h, 200×) and relatively brisk mitoses. Black arrows indicated mitotic figures (i, 400×). Multifocal tumor necrosis was observed (j, 200×). Immunohistochemical staining for Ventana IHC ALK (clone D5F3) demonstrated diffuse expression (k, 200×). In the hotspot area, the Ki‐67 proliferation index was 30% (l, 200×).

The tumor was relatively large in volume, and there was no evidence of metastatic disease within the chest or abdomen. A multidisciplinary team recommended performing a biopsy for a definitive pathological diagnosis. A tumor biopsy was performed, and a pathological examination revealed a neoplastic lesion with extensive necrosis (Figure 2a). Microscopically, the tumor was composed of spindle cells admixed with infiltrating inflammatory cells, closely resembling an IMT (Figure 2b). The tumor cells expressed S100 (Figure 2c), CD34 (Figure 2d), ALK‐1A4 (Figure 2e), and EMA (focal and weak). They were negative for PCK, SOX10, NTRK, LCA, SMA, MyoD1, myogenin, CD99, Syn, TdT, CD38, and STAT6. The average Ki‐67 index was 15%.

**FIGURE 3:**
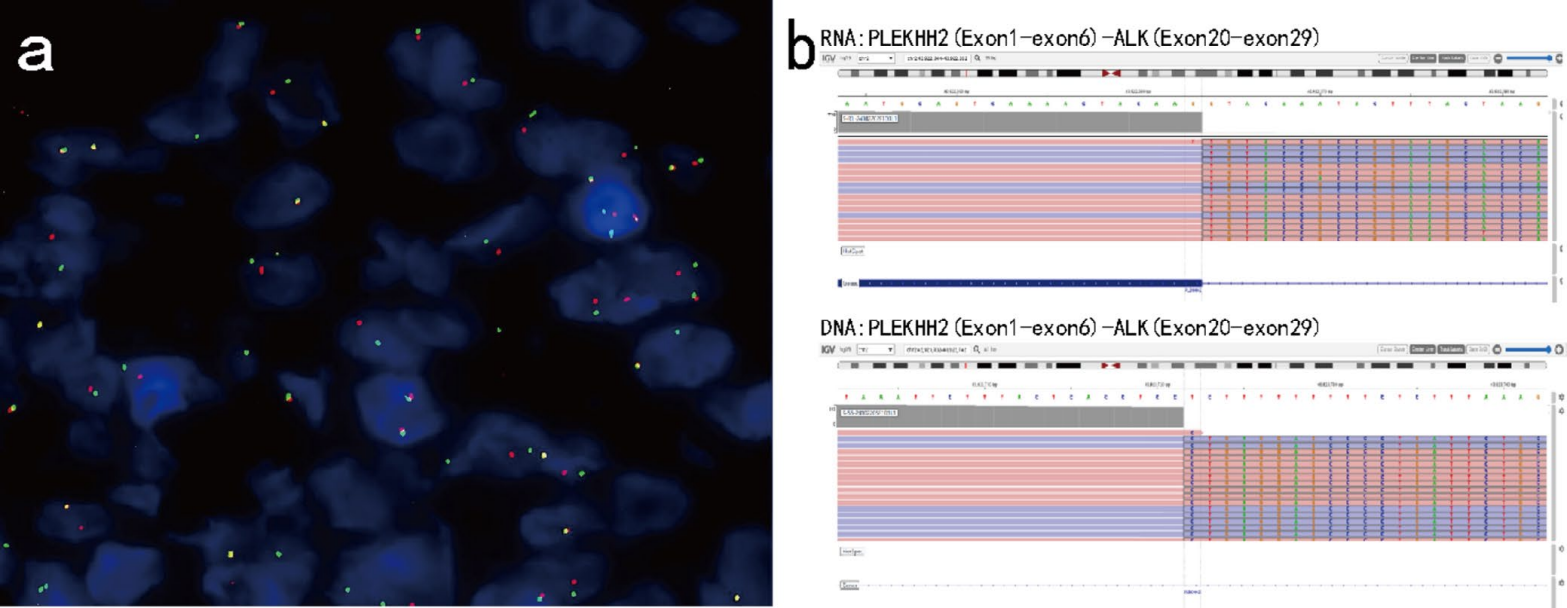
Molecular characteristics of the tumor. Break‐apart fluorescence in situ hybridization verified the ALK receptor tyrosine kinase gene (*ALK*) rearrangement (a), and DNA‐ and RNA‐based NGS confirmed the *PLEKHH2*::*ALK* fusion (b).

To clarify the mutation status of the *ALK* gene, the tumor tissue obtained by biopsy was evaluated by break‐apart fluorescence in situ hybridization (FISH). One hundred nonoverlapping cells were scored, and more than 20% of the tumor cells with abnormal signals were considered positive for gene rearrangement. The FISH results confirmed the *ALK* rearrangements in our patient (Figure 3a). To confirm the fusion site, next‐generation sequencing (NGS) was performed on the Ion Torrent platform (DA8600), which revealed that the patient had an *ALK* gene fusion: *PLEKHH2* Exon 6::*ALK* Exon 20 (Figure 3b). The combined histomorphological, immunophenotypic, and molecular findings led to the diagnosis of an ALK‐positive mesenchymal tumor.

Following multidisciplinary discussion and evaluation, the patient underwent video‐assisted thoracic surgery for left lung lobectomy with left mediastinal lesion and regional mediastinal lymph node dissection on June 3, 2024. Microscopic examination revealed a heterogeneous neoplasm. The majority of tumor cells exhibited a growth pattern characterized by diffuse sheets and focal infiltrative proliferation, predominantly composed of spindled to ovoid neoplastic cells embedded in myxomatous stroma (Figure 2f). In focal areas, the tumor cells displayed mild cytological atypia, accompanied by rare mitotic figures (MFs) and focal necrosis. Abundant hyalinized blood vessels and focal hyalinized stroma were present (Figure 2g). In some areas, tumor cells had increased density with moderate to severe pleomorphism (Figure 2h), and the MFs were plentiful (approximately 20 MFs/10 high‐power fields [HPFs]) (Figure 2i) with multifocal tumor necrosis (Figure 2j). According to the Federation of Cancer Centers Sarcoma Group (FNCLCC) grading, the morphology of the neoplasm was intermediate–high grade.

A panel of immunohistochemical stains was performed for further characterization, and the immunohistochemical results were consistent with the biopsy specimen findings. Furthermore, validation with Ventana IHC ALK (clone D5F3) demonstrated diffuse ALK protein positivity in tumor cells (Figure 2k). In the hotspot area, the Ki‐67 proliferation index was 30% (Figure 2l).

**FIGURE 4:**
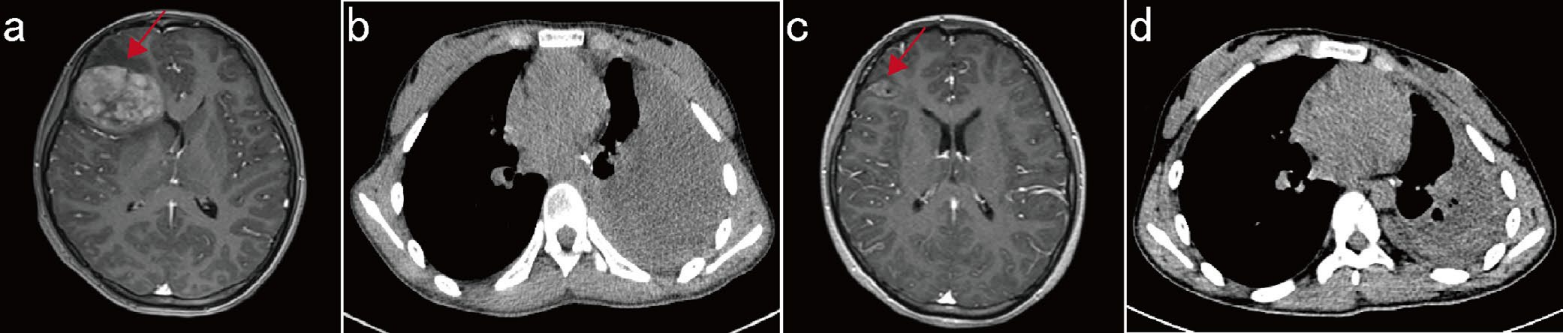
Radiographic images before and after the initiation of ALK‐targeted therapy. Cranial MRI (2024/9/14) revealed a 53 × 37 mm cranial mass (a), and a heterogeneous, slightly hyperdense mass in the left thoracic cavity was detected via a CT scan (b). After 1 month of alectinib therapy, imaging examinations revealed an obvious decrease in the size of the brain metastasis (c). The chest lesion was significantly smaller in size (d).

The patient recovered well after surgery and was discharged on June 13, 2024. However, 3 months after surgery, the patient was readmitted with chest tightness, cough, headache, and vomiting for 1 week. A cranial MR scan revealed a brain metastasis in the right frontal lobe (maximum diameter, 53 mm; Figure 4a), and a chest CT scan revealed a heterogeneous, slightly hyperdense mass in the left thoracic cavity, suggestive of tumor recurrence at the original site after surgery (Figure 4b). The therapeutic outcome was considered progressive disease (PD). Given that the tumor was positive for an *ALK* rearrangement, the patient was started on alectinib in September 2024. One month after the initiation of alectinib therapy, a cranial MR scan revealed an obvious decrease in the size of the brain metastasis, and its maximum diameter was reduced to 24 mm (Figure 4c). Compared with previous imaging, a chest CT scan revealed a decrease in the extent of the mass in the left thoracic cavity (Figure 4d). The disease response was considered a partial response (PR) according to the Response Evaluation Criteria in Solid Tumors (RECIST) version 1.1 after targeted therapy. The patient still received alectinib therapy, and no evidence of disease progression was found during the next 6‐month follow‐up.

## 3 Discussion

Here, we describe a rare case of an *ALK*‐rearranged mesenchymal tumor that occurred in the left thoracic cavity and involved the lower lobe of the left lung. Although the differential diagnosis of the S100‐positive spindle cell neoplasm included malignant peripheral nerve sheath tumor (MPNST), several features argued against it. The tumor exhibited strong diffuse CD34 expression, which is unusual in high‐grade MPNST as only 9% of cases show such diffuse reactivity [26]. This, combined with SOX10 negativity (SOX10 is a moderately sensitive but very specific marker for MPNST [27]) and the lack of characteristic morphology such as alternating hypercellular and myxoid areas, collectively argued against this diagnosis.

In our case, the tumor's histological features suggest that the neoplasm had overtly malignant features and may be associated with an aggressive clinical course. This might also partly account for the occurrence of distant tumor metastasis in patient 3 months after surgery. At present, no clinicopathological variables have been shown to be related to the prognosis of *ALK*‐rearranged mesenchymal neoplasms, although a previous study suggested that tumors in deep locations with high‐grade features may follow a more aggressive clinical course [3]. From a practical perspective, it is advisable to histologically grade *ALK*‐rearranged soft tissue tumors, as is done with mesenchymal tumors harboring fusions with other kinases. Tumors with fibrosarcoma‐like or diffuse epithelioid features, particularly those associated with elevated mitotic activity or tumor necrosis, should be classified as high grade [2].

Previous studies of *ALK*‐rearranged mesenchymal neoplasms have identified diverse *ALK* fusion gene partners, including *AK5*, *CLIP1*, *CLTC*, *DCTN1*, *EML4*, *ERC1*, *FLNA*, *FMR1*, *HMBOX1*, *KLC1*, *MYH10*, *PLEKHH2*, *PRRC2B*, *PPP1CB*, *STRN*, *TIMP3*, *TPM3*, and *VCL* [2]. In our study, the tumor was identified to harbor the *PLEKHH2*::*ALK* fusion gene. The *PLEKHH2* gene (2p21) encodes an intracellular protein highly enriched in renal glomerular podocytes that plays a structural and functional role in the podocyte foot processes. The presence of an α‐helical coiled‐coil domain was observed in the N‐terminus of *PLEKHH2* [4]. To date, no obvious relationship between the *ALK* fusion partner type and morphological patterns in *ALK*‐rearranged sarcomas has been noted. The identification of *ALK* fusions in patients with aggressive or unresectable tumors raises the possibility of targeted therapy with ALK inhibitors, such as crizotinib, alectinib, ceritinib, and brigatinib [22, 23]. However, the number of available patients who have undergone targeted ALK therapy for an *ALK*‐rearranged mesenchymal neoplasm is relatively limited in the literature. Zhao et al. reported that a patient developed lung metastases 60 months after the initial surgery. Following resection of the metastatic pulmonary tumors, the patient developed gastric metastases at the 96‐month follow‐up, after which he was treated with a second resection followed by targeted therapy with ensartinib (a second‐generation ALK inhibitor) for 5 months. The patient was alive without disease at the last 130‐month follow‐up [2]. This report suggests that adjuvant ALK‐targeted therapy shows better therapeutic efficacy; however, there is less evidence in the neoadjuvant setting. Recently, Coppock et al. reported a 21‐year‐old woman with a 15.7 × 13.5 × 11.8 cm heterogeneously enhancing pelvic mass of indeterminate origin with focal cystic changes. CT‐guided biopsy was performed, and the pathology results revealed a histomorphologically heterogeneous mesenchymal neoplasm expressing both S100 and CD34, with a subsequently identified *PLEKHH2*::*ALK* fusion. Presurgical therapy was initiated with the ALK inhibitor brigatinib, 90 mg orally daily for 12 weeks. The patient tolerated neoadjuvant brigatinib quite well, with a dramatic reduction in the tumor volume, allowing for less extensive surgery [14]. Similarly, the tumor volume was also relatively large, and *PLEKHH2*::*ALK* gene fusion was detected in this patient. Three months after surgery, brain metastasis occurred, and the patient subsequently demonstrated a significant response to the ALK inhibitor. This raises the important issue that neoadjuvant therapy with ALK inhibitors is much more likely to result in a significant reduction in the size of the mass, improve the probability of complete surgical removal, and reduce the risk of metastasis after surgical management. Owing to the relatively small number of cases, further follow‐up studies are needed to elucidate the long‐term prognosis of *ALK*‐rearranged mesenchymal neoplasms.

In summary, we reported an uncommon *ALK*‐rearranged mesenchymal tumor occurring in the left thoracic cavity and involving the lower lobe of the left lung. Because of their rarity as well as their diverse morphologies and nonspecific immunoprofiles, the histological diagnosis of *ALK*‐rearranged mesenchymal neoplasms is often challenging. Therefore, when a pathologist encounters a mesenchymal tumor, particularly in the pediatric age group, this should prompt further testing for *ALK* alterations by FISH or NGS after ruling out entity tumors. The success of this diagnostic, therapeutic, and interventional sequence highlights the importance of preoperative biopsy and molecular testing to establish a diagnosis before treatment.

## 4 Conclusion

We report a case of a giant *PLEKHH2*::*ALK*‐rearranged mesenchymal neoplasm that occurred in the left thoracic cavity, involved the lower lobe of the left lung, and exhibited overtly malignant features. The rapid recurrence and metastasis of the tumor after the operation suggested that its aggressive biological behavior was related to the histological grade. This should be highly important to pathologists and clinicians. Moreover, the detection of *ALK* fusions in these neoplasms is highly important not only from a diagnostic perspective but also from a therapeutic standpoint, as some cases may behave aggressively. Future investigations are needed to clarify the optimal timing of targeted therapy and its potential clinical feasibility in the neoadjuvant setting.

## Author Contributions

X.N., J.F., and X.D. designed the study. B.H., J.H., and D.L. performed molecular testing and analyzed the data. S.G. and J.W. drafted the manuscript. H.S. provided imaging data. X.C. helped to revise the manuscript. All authors contributed to the article and approved the submitted version.

## Funding

This work was supported by the National Key Research and Development Program of China (No. 2022YFF1203300), the National Natural Science Foundation of China (No. 82203333), and the Natural Science Foundation of Hubei Province (No. 2022CFB078 and 2023AFB986).

## Ethics Statement

The study involving a human participant was reviewed and approved by the Institutional Review Board of Wuhan Union Hospital and Huazhong University of Science and Technology (Ethical Approval Number: S‐377). Written informed consent for participation in this study was obtained from the patient's legal guardian (the patient's mother).

## Consent

Written informed consent was obtained from the patient's legal guardian (the patient's mother) for the publication of any potentially identifiable images or data included in this article.

## Conflicts of Interest

The authors declare no conflicts of interest.

## Data Availability

The data that support the findings of this study are available from the corresponding author upon reasonable request.
